# Polymorphic transitions in flufenamic acid-trehalose composites

**DOI:** 10.1016/j.ijpx.2023.100200

**Published:** 2023-07-23

**Authors:** Yuying Pang, Simon Gaisford, Oxana V. Magdysyuk, Gareth R. Williams

**Affiliations:** a UCL School of Pharmacy, University College London, 29-39 Brunswick Square, London WC1N 1AX, United Kingdom; b Diamond Light Source, Harwell Science and Innovation Campus, Didcot, Oxfordshire OX11 0DE, United Kingdom

**Keywords:** Solid Dispersions, Flufenamic acid, Trehalose, Synchrotron X-ray diffraction, Differential Scanning Calorimetry

## Abstract

The combination of poorly-soluble drugs with small molecule co-formers to generate amorphous solid dispersions (ASDs) has great potential to improve dissolution rate and kinetic solubility, and thus increase the bioavailability of these active ingredients. However, such ASDs are known to be unstable and to crystallise upon storage or heating. In this work, we explore the crystallisation of flufenamic acid (FFA) from ASDs prepared with trehalose. FFA-trehalose mixtures were prepared at a range of *w*/w composition ratios, heated to melting and crash cooled to form ASDs. They were then subject to a further heat/cool cycle, which was monitored by simultaneous differential scanning calorimetry – X-ray diffraction to observe the phase changes occurring. These varied with the composition of the blend. Upon short-term storage, formulations with low trehalose contents (FFA:trehalose 5:1 *w*/w) recrystallised into form I FFA, while higher trehalose contents crystallised to FFA form IV. When heated, all FFA trehalose combinations ultimately recrystallised into form I before melting. Upon a second cooling cycle, systems with low trehalose content (FFA:trehalose 5:1 *w*/w) recrystallised into form IV, while higher trehalose contents led to FFA form I. It is thus clear that even with a single excipient it is possible to control the crystallisation pathway through judicious choice of the formulation parameters.

## Graphical abstract

**Figure:**
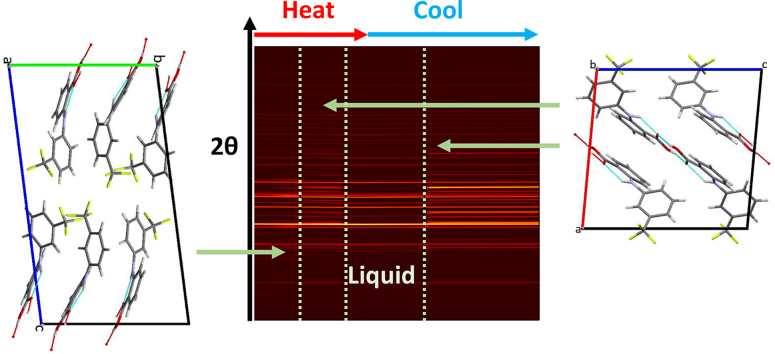

## 1 Introduction

Many active pharmaceutical ingredients (APIs) suffer from poor aqueous solubility, with ca. 75% of new small molecule drugs in development pipelines falling in Class II or IV of the Biopharmaceutical Classification System (BCS). One option to overcome this issue is to use amorphous systems (Attia et al., 2023; Kumari et al., 2023). However, the amorphous form is thermodynamically unstable and even when APIs are blended with stabilising excipients to form amorphous solid dispersions (ASDs) there is a risk of crystallisation. The latter is a complicated process during which a number of polymorphs may co-exist within the same formulation (Aher et al., 2023; Grooff et al., 2007; Jarrells and Munson, 2022; Maniruzzaman et al., 2013; Meng et al., 2015).

Polymers have been frequently employed to inhibit crystallisation and increase the stability of amorphous materials (Baird and Taylor, 2012; Maniruzzaman et al., 2013; Van den Mooter, 2012; Wegiel et al., 2013). An alternative approach to stabilise ASDs and alter crystallisation kinetics is to use small-molecule additives (Davey et al., 2002). Studies on paracetamol indicate that surfaces such as glass and silica help to crystallise and stabilise metastable form III if the heating and cooling rate are carefully controlled (Burley et al., 2007; Di Martino et al., 1997; Ehmann and Werzer, 2014; Nanubolu and Burley, 2012). The addition of lactose and trehalose result in incomplete vitrification after melting and crash cooling, and the presence of residual paracetamol form II in the mixture. However, paracetamol form III grew in from the mixture containing lactose while with trehalose form II crystallised and then partially converted to form I (Ehmann and Werzer, 2014; Telford et al., 2016). Hence, saccharides appear to have the ability to selectively stabilise polymorphs. This is thought to arise via intermolecular interactions between the API crystal surface and the excipients (Dowling et al., 2010; Gift et al., 2008; Hiremath et al., 2004; Lee et al., 2006; Staab et al., 1990). Preferential interactions between an additive and a metastable phase may result in stabilisation of the latter, or alter the balance of growth rates of different polymorphic forms (Dowling et al., 2010; Kim and Choi, 2002).

**Fig. 1:**
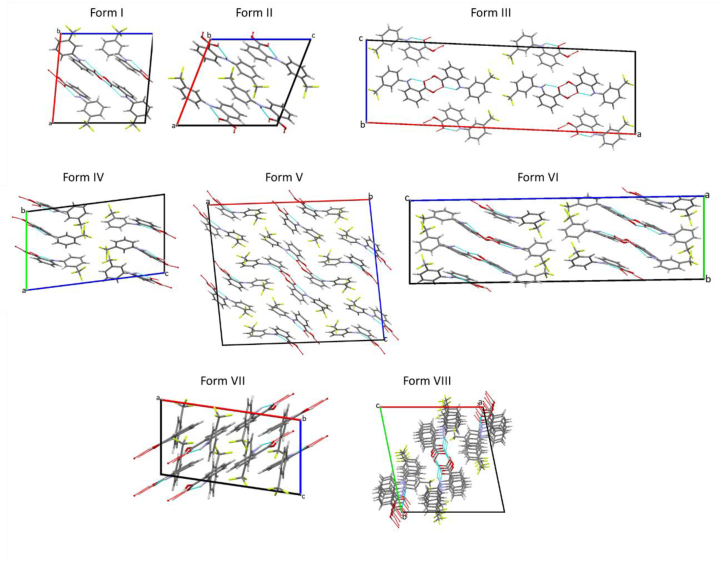
The molecular arrangements in the various forms of FFA. Structures are taken from the Cambridge Structural Database, with reference codes as follows: form I, FPAMCA11; form II, PAMCA17; form III, FPAMCA; form IV, FPAMCA15; form V, FPAMCA16; form VI, FPAMCA14; form VII, FPAMCA12; form VIII, FPAMCA13. Disorder present in form IV has been omitted for clarity.

In this work, we explore crystallisation and interpolymorph conversion in composites of flufenamic acid (FFA; 2-[3-(trifluoromethyl)amino]benzoic acid) and trehalose. FFA is a non-steroidal anti-inflammatory drug (NSAID) which falls in Class II of the BCS and thus suffers from low solubility. To overcome this limitation, amorphization has been explored (Vasconcelos et al., 2007). FFA is also known to be highly polymorphic, with eight structurally characterised forms reported (Delaney et al., 2014). These are illustrated in Fig. 1. The reported melting points for the polymorphs are all very similar, lying between 120 and 135 °C and thus the stability order is not well established (Lopez-Mejias et al., 2012).

We have previously shown that heating a glass of pure FFA results in an initial recrystallisation into an unknown polymorph at ca. 54 °C, and that this then converts to form IV and then to form I (Pang et al., 2022). The behaviour is very different when combining FFA with ethyl cellulose (EC) and hydroxypropylmethylcellulose (HPMC). Quench cooling of the mixtures results in the formation of ASDs which, upon heating, display crystallisation pathways dependent on both the polymer viscosity and chemistry (Pang et al., 2022). FFA form IV is found to be stabilized by HPMC 6 and 100,000 cp and EC 4 cp, all of which inhibit the from IV to I conversion upon heating. Polymorphic transitions are also inhibited when the polymer content of the ASD is increased, and with a 1:5 *w*/w FFA-polymer blend the drug remained mostly amorphous upon heating. Both the viscosity and substitution pattern of HPMC are found to influence the recrystallisation process. It is thus clear that there are myriad and subtle effects involved in the recrystallisation from polymer-drug ASDs. Here, we sought to expand on our previous study to explore the effects of using small-molecule excipients for ASD preparation.

Trehalose is a nonreducing disaccharide in which two glucose units are linked in a α-1,1-glycosidic linkage (α-d-glucopyranosyl-α-d-glucopyranoside). It has a high glass transition temperature, and is widely utilised in the food and pharmaceutical industries (Peng et al., 2017). Given its ability to form hydrogen bonds, we hypothesised that trehalose could be a suitable co-former to generate FFA ASDs. A series of ASDs were generated by quench-cooling, and the crystallisation and polymorphic phase transitions studied in detail using differential scanning calorimetry with in-situ synchrotron diffraction.

## 2 Materials and methods

### 2.1 Materials

Flufenamic acid (form I) and trehalose (D-trehalose anhydrate) were purchased from Acros.

### 2.2 Methods

#### 2.2.1 Hyphenated differential scanning calorimetry-X-ray diffraction (DSC-XRD)

Appropriate amounts of FFA and trehalose were weighed in 5 ml glass vials to give final mass ratios of 1:2, 1:1, 3:2 and 5:1 (*w*/w; FFA:trehalose). The samples were then mixed for ca. 3 min using a vortex mixer. Ca. 5–10 mg of sample was heated from 0 °C to 150 °C at 10 °C/min and then cooled to 0 °C in a TA Instruments Q2000 DSC to produce amorphous solid dispersions. The obtained solids were stored in a fridge (6 °C) before characterisation by DSC-XRD. DSC measurements were performed with a modified Q20 DSC (TA Instruments), with the instrument modification, experiment set up and data analysis protocols being the same as described previously (Clout et al., 2020; Pang et al., 2022). The DSC was mounted and aligned on the sample stage in the experimental hutch of Beamline I12 (JEEP) (Drakopoulos et al., 2015) at the Diamond Light Source to allow a monochromated X-ray beam (0.5 mm × 0.5 mm; λ = 0.234 Å) to pass through holes in the DSC cell. A Thales Pixium RF4343 detector was located 1.9 m behind the sample. The DSC was calibrated with an indium standard and the Pixium detector with cerium dioxide, prior to experiments beginning. The quench cooled products were reheated to 160 °C at 10 °C/min, and then air cooled to ca. 60 °C.

Diffraction patterns (5 s, with a 1 s pause between each) were collected using the Diamond Generic Data Acquisition (GDA) software. The resultant two-dimensional Pixium images were masked, and processed using the DAWN Science Workbench software into one-dimensional diffraction patterns by azimuthal integration (Filik et al., 2017). TOPAS-Academic V5 was then applied to analyze selected patterns with the Rietveld method (Coelho, 2018). This resulted in realistic values for the unit cell parameters at elevated temperatures. FFA polymorph structures were obtained from the Cambridge Structural Database (CSD). Batch Rietveld refinements were finally performed to determine phase fractions as a function of temperature.

#### 2.2.2 Fourier transform infrared (FTIR) spectroscopy

FTIR spectroscopy was conducted with a Perkin Elmer Spectrum 100 instrument. All spectra were recorded between 650 and 4000 cm^−1^ with 64 scans at a resolution of 4 cm^−1^. FFA, trehalose and the samples explored in DSC-XRD were all evaluated. The drug/trehalose mixtures were heated in the Q2000 DSC from 40 °C to 150 °C at 10 °C/min before equilibrating back to 0 °C and then reheated to the temperature of interest (after each recrystallisation event) at 10 °C/min. The samples were then removed from the DSC and characterised by FTIR.

## 3 Results and discussion

FFA/trehalose (FFA/T) blends were heated to melting and then cooled to 0 °C in a Q2000 DSC to produce amorphous materials. The DSC traces suggest that no recrystallisation took place during the cooling processes (Supplementary Information, Fig. S1). The solids obtained were stored in the fridge before characterisation by DSC-XRD at the Diamond Light Source (< 5 days later). This was expected to minimise the extent of recrystallisation. However, from the XRD results it is clear that there are some reflections in the contour plots at the beginning of the DSC-XRD experiments, which suggests some degree of recrystallisation occurred during storage.

### 3.1 1:2 *w*/w FFA/trehalose

**Fig. 2:**
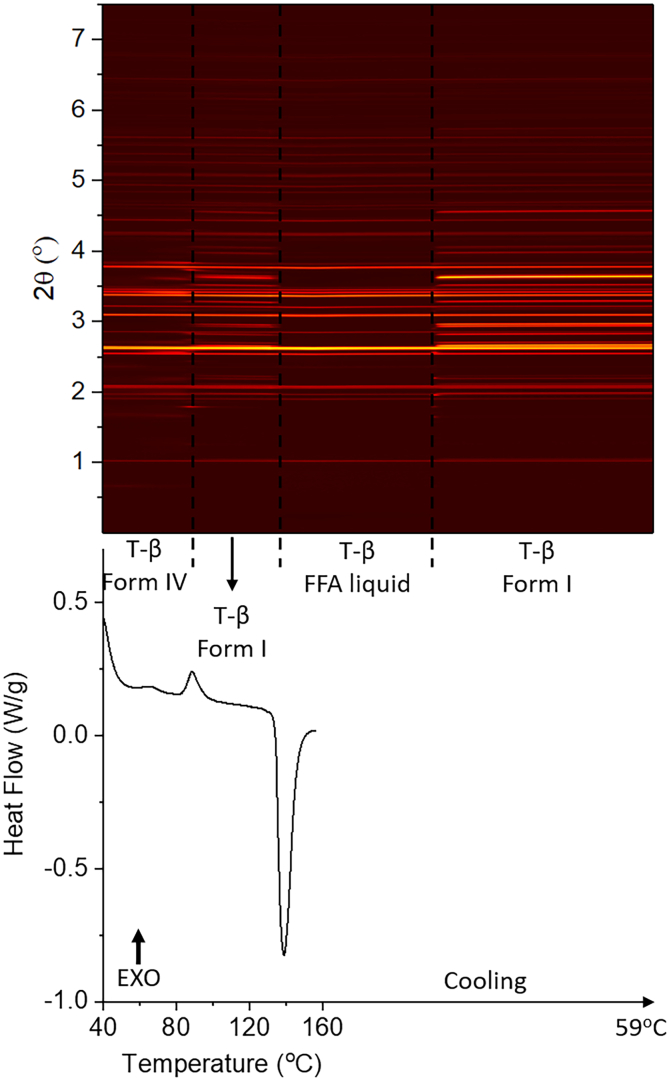
DSC-XRD data for the reheating and cooling of a FFA/T blend prepared at 1: 2 w/w. T-β: trehalose β.

DSC-XRD data for a 1:2 FFA:trehalose weight ratio mixture (FFA/T 1:2 *w*/w) are presented in Fig. 2. A small exotherm is observed in the DSC at ca. 65 °C, which is followed by a more distinct exothermic event with onset at 84 °C. The latter is coincident with with changes in the positions of Bragg reflections in the XRD data. Following this, there is an endothermic peak with onset at 135 °C, which is reported to be the melting point of FFA form I (Lopez-Mejias et al., 2012). However, instead of a total loss of Bragg reflections after melting, a change of diffraction pattern is shown in the XRD profile, and thus some crystalline material (presumably trehalose) remains. Finally, the diffraction pattern changes again upon cooling, which suggests recrystallisation.

Phase identification was carried out against patterns recorded at 78 °C, 115 °C, 121 °C (during heating) and 75 °C (during cooling) (Fig. S2, Table S1) using the Rietveld method. The pattern recorded at 78 °C comprises FFA form IV and trehalose β. This suggests FFA in the sample recrystallised into form IV during storage, and trehalose crystallised into its β form. Refinement against the pattern at 115 °C confirms that the solid is FFA form I and trehalose β. The pattern recorded at 121 °C during cooling is entirely trehalose β. Thus, during heating, FFA converts from form IV to form I and then form I melts at 135 °C. Trehalose remains in its β form throughout the experiment. During cooling, Rietveld refinement reveals that the pattern collected at 75 °C is FFA form I and trehalose β, which means in this formulation molten FFA recrystallises into form I upon cooling.

**Fig. 3:**
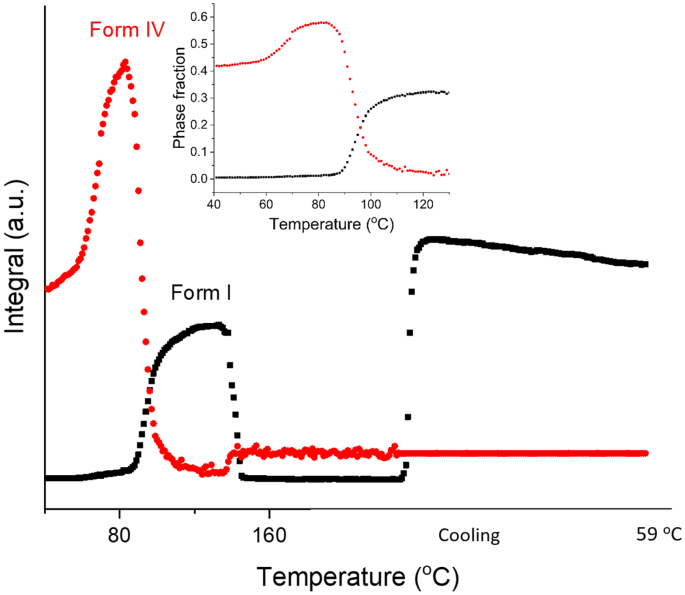
Plot of integrated total diffracted intensity as a function of temperature for FFA form IV and form I in a FFA/T 1: 2 *w*/w blend.

In order to explore the presence of the different FFA polymorphs as a function of temperature, batch refinement was carried out and the results are plotted in Fig. 3. At 40 °C the FFA in the sample is present as form IV. The amount of form IV increases with heating, with a sharp increase in the crystallisation rate at ca. 65 °C (believed to be the origin of the small initial exotherm in the DSC data). This continues until the tempeature reaches 84 °C, as more drug precipitates out from the amorphous phase. After 84 °C, where there is a marked exotherm in the DSC data, the amout of form IV decreases and form I FFA begins to grow in.

In Fig. 3 (inset) the phase fraction curves for FFA form IV and I are seen to cross at around 15% of the FFA present, which is less the half the maximum quantity of form I. Moreover, the phase fraction of form I at 120 °C is lower than the maximum percentage of form IV. These observations together suggest that not all the form IV converts to form I during the polymorphic transition (84–120 °C); instead some of it turns amorphous (some form IV may melt). A linear fit was carried out at the straightest section of the curves, at around the point at which they cross (91-98 °C). The rate of decay of form IV and the growth of form I are −2.32 °C^−1^ and 0.94 °C^−1^ respectively, with form I growing in more slowly then form IV disappears. During heating, some form IV converts to form I through a polymorphic transiton, while some becomes amorphous. The conversion of crystalline material to an amorphous phase clearly occurs more rapidly than the molecular rearrangements needed to form I. Thus, the decay of form IV happens faster than the growth of form I. The growth of form I peaks at 125 °C, after which the amount present decreases rapidly to a minimum at 145 °C. Both form I and IV stay at a minimum level before form I starts to grow back in during air cooling. The amount of form IV appears to increase at 140 °C, but this is an artefact arising because at this temperature FFA turns into a liquid. During batch refinement, the crystal structures of form I and IV are used as references to calculate the phase fractions and integral intensities. There is a fully amorphous diffraction pattern at temperatures above 140 °C, which means that it is not possible to effectively fit the structures to the datasets and thus the phase fractions calculated are not fully accurate.

### 3.2 1:1 w/w FFA/trehalose

**Fig. 4:**
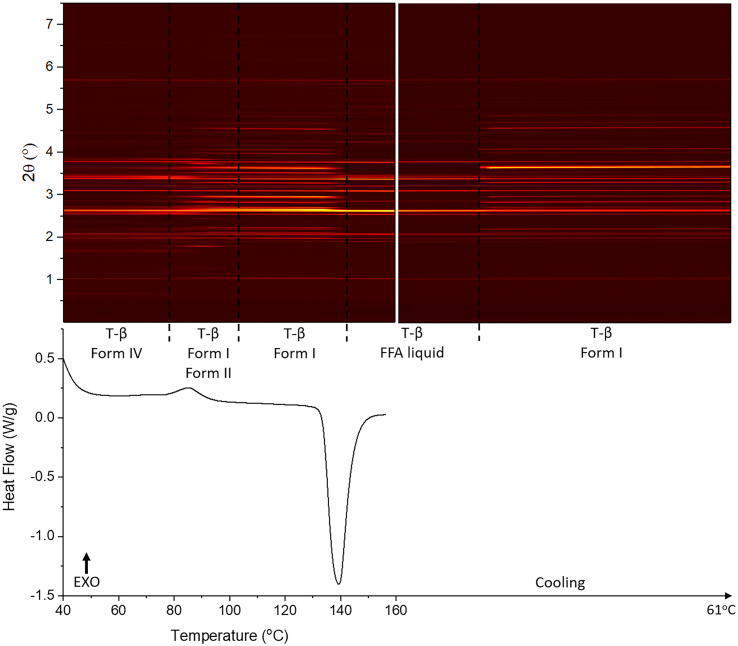
DSC-XRD data for the reheating and cooling of a 1: 1 w/w FFA/T blend. T-β: trehalose β.

DSC-XRD data for the mixture with a FFA:trehalose ratio of 1:1 w/w (FFA/T 1:1 *w*/w) are given in Fig. 4. The DSC data show an exothermic peak with onset at 78 °C, with concomitant changes seen in the diffraction pattern. At about 100 °C, the reflection pattern changes slightly, but no events are observed in the DSC data. Following this there is an endothermic event with onset at 135 °C; similar to the observations with the FFA/T 2:1 w/w sample some reflections disappear at this point while those from trehalose remain, as a result of crystalline trehalose being present after all the FFA has melted. Finally, the diffraction pattern changes again during the cooling process, which suggests that there is recrystallisation.

As previously, phase identifications were carried out using Rietveld refinement: patterns at 47 °C, 91 °C, 128 °C, 157 °C (during heating) and 68 °C (cooling) were examined individually (Fig. S3, Table S2). The initial material (47 °C) comprises FFA form IV and trehalose β (Fig. S3). As for the FFA/T 1: 2 w/w sample, FFA/T 1: 1 w/w recrystallised into FFA form IV and trehalose β during storage. The refinement against the pattern at 91 °C shows that the solid is a mixture of form I and form II FFA and trehalose β. The pattern recorded at 128 °C after the exothermic event comprises form I FFA and trehalose β. At 157 °C, the sample is identified as being entirely trehalose β. Thus, during heating FFA converts from form IV to form I/II. It appears that the form II generated subsequently converts to form I, which then melts at 135 °C while trehalose remains in its β form. Refinement at 68 °C, recorded on cooling, shows the sample to comprise FFA form I and trehalose β. Here again the FFA recrystallises into form I on cooling.

**Fig. 5:**
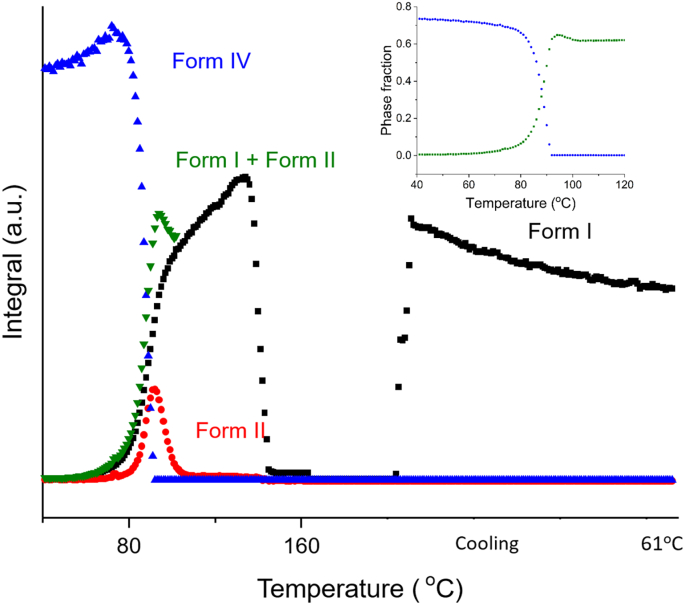
Plot of integrated total diffracted intensity for FFA form IV, II and I as a function of temperature in a 1: 1 *w*/w FFA: trehalose mixture.

Batch refinement was performed on the full set of XRD data, and Fig. 5 shows the amount of each FFA polymorph present in the sample as a function of temperature. FFA is present as form IV from the start of the experiment to ca. 77 °C, after which the amount of form IV decreases while form I and form II begin to grow in. This change coincides with the onset of the exothermic event in DSC. This trend continues until the temperature reaches 91 °C. The amount of form II decreases from 91 to 100 °C, while the amount of form I present keeps increasing until its melting point at 133 °C. Thus, over the temperature range from 77 to 100 °C (exothermic event), the solid experiences a form IV to I and II and form II to I transition. During the cooling process, form I starts to grow in again.

In Fig. 5 (inset) the phase fraction curves can be seen to cross at around half of the maximum quantity of form IV. The total phase fraction of form I and II at 100 °C (after the polymorphic transition) is a little lower than that of form IV at the start of the experiment, suggesting that not all form IV converts to form I and II after the transition; instead, some of it turns amorphous. A linear fit was carried out at the straightest section of the curves, at around the point at which they cross (86-91 °C). The rate of decay of form IV and the growth of forms I and II are −1.49 °C^−1^ and 0.69 °C^−1^, respectively. Similarly to the 1:2 data in Fig. 3, the amount of form IV decreases faster than the total rate of growth of forms I and II. This again suggests that some form IV turns amorphous. Additionally, the growth rates of form I (0.38 °C^−1^) and form II (0.32 °C^−1^) are almost the same, suggesting that form IV converts to form I and II at the same speed.

### 3.3 3:2 w/w FFA/trehalose

**Fig. 6:**
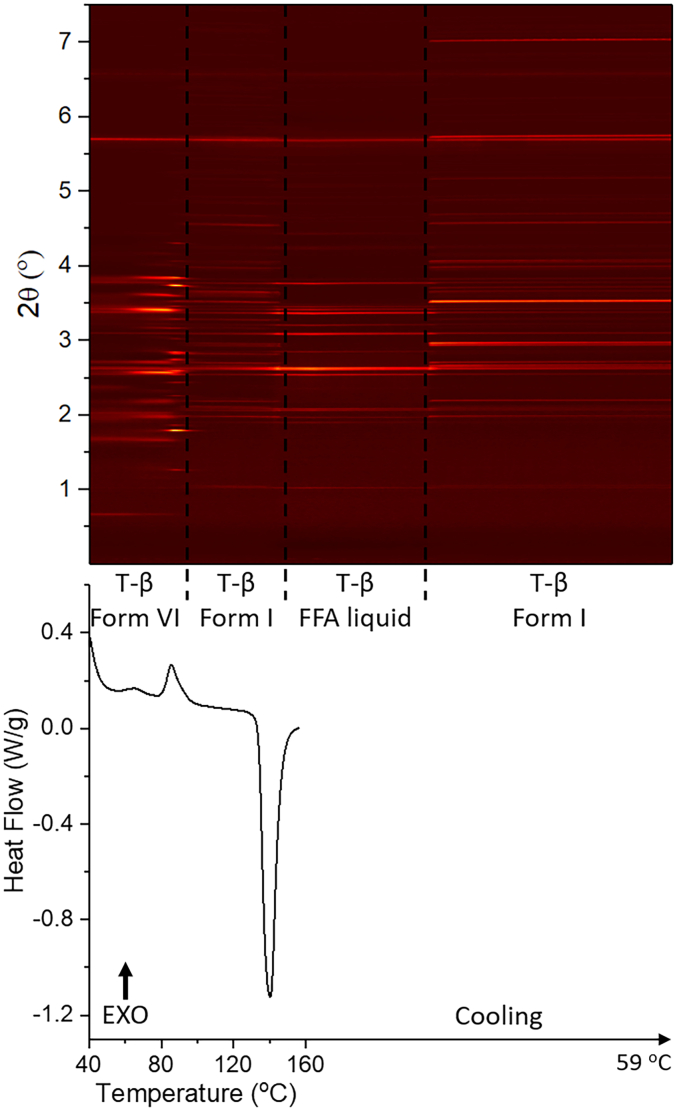
DSC-XRD data for the reheating and cooling of a3: 2 *w*/w FFA/T blend. T-β: trehalose β.

The mixture comprising FFA:trehalose 3:2 w/w shows the same phase transitions as the 1:2 w/w analogue (Fig. 6). A small exothermic event is seen at ca. 65 °C followed by a larger exotherm with onset at 80 °C is observed, coincident with a distinct shift in the positions of the Bragg reflections in the XRD data. Following this, there is an endothermic peak with onset at 135 °C, but crystalline material remains beyond this point. Finally, the diffraction pattern changes again during cooling, consistent with recrystallisation.

Phase identifications were carried out with Rietveld refinement against patterns recorded at 71 °C, 120 °C, 160 °C (during heating) and 63 °C (during cooling). Analogous findings to those obtained with other FFA/T blends are observed. Selected XRD patterns and refinement parameters are shown in Fig. S4 and Table S3.

**Fig. 7:**
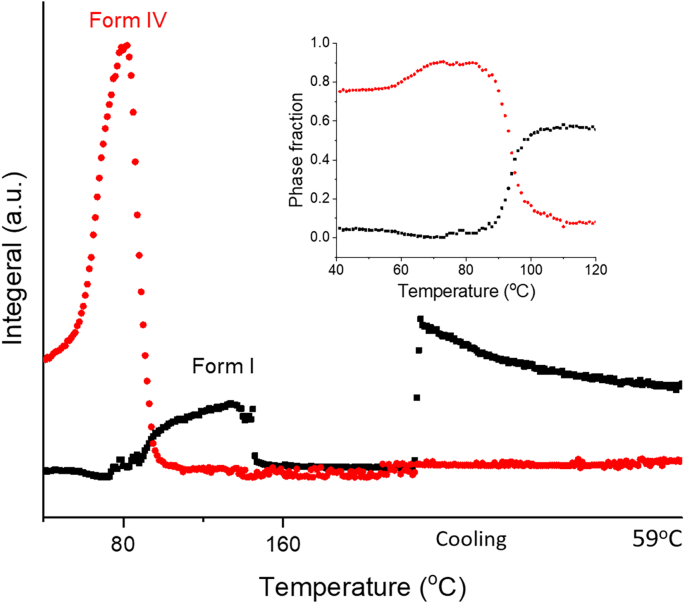
Plot of integrated total diffracted intensity for FFA form IV and form I as a function of temperature in the FFA: trehalose 3: 2 *w*/w blend.

The relative proportions of FFA polymorphs were analysed by batch refinement (Fig. 7). This reveals the growth of form IV before 80 °C, with a rapid increase in the rate of growth at around 65 °C. This suggests that the starting material comprises a mixture of form IV and amorphous FFA, and that amorphous FFA converts to form IV during heating. After this a transition from form IV to I occurs before melting. The recrystallisation of form I during cooling is also observed. A linear fit was carried out at the straightest section of the curves around the point at which they cross (94–103 °C). The rates of decay of form IV and growth of form I are −0.03 °C^−1^ and 0.01 °C^−1^, respectively with form I growing more slowly. The phase fraction of form I at 100 °C (after the polymorphic transition) is somewhat lower than the maximum percentage of form IV, as previously suggesting that not all form IV converts to form I. During the cooling process, there are only form I appears in the sample.

### 3.4 5:1 w/w FFA/trehalose

**Fig. 8:**
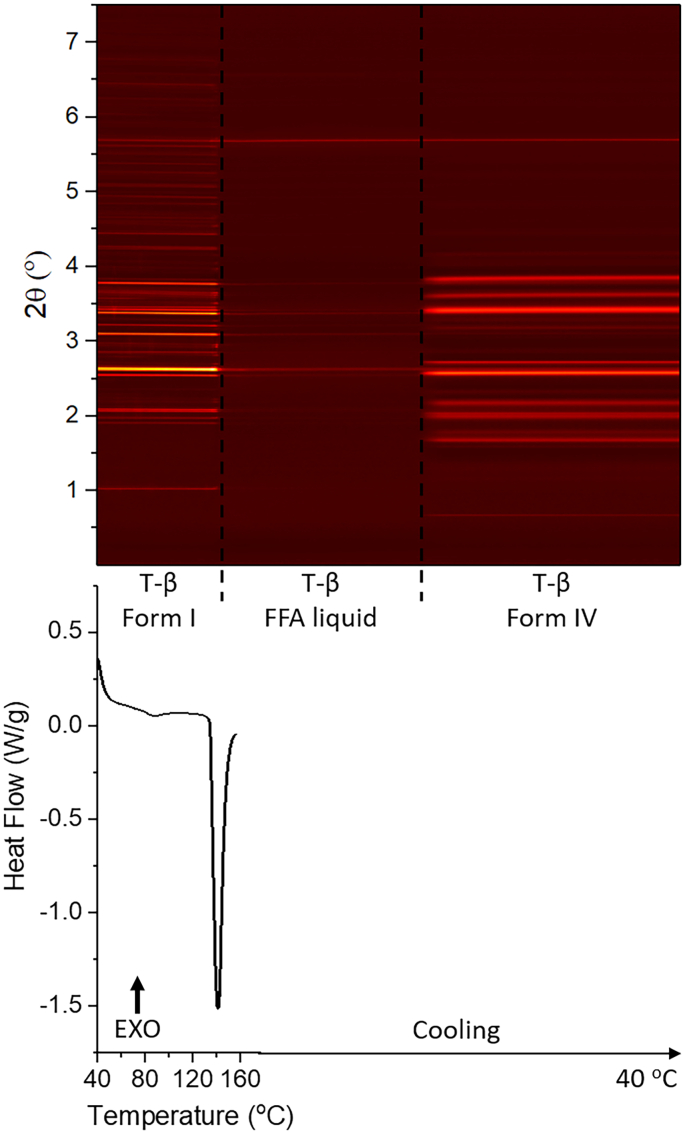
DSC-XRD data for the reheating and cooling of a 5: 1 *w*/w FFA/T blend. T-β: trehalose β.

The FFA:trehalose 5:1 *w*/w dispersion gives a different phase transition profile to the previous mixtures explored (Fig. 8). Only a single endothermic peak at 141 °C (peak temperature) can be seen in the DSC profile, together with the total loss of diffraction pattern on the contour plot, which suggests that this sample recrystallised into form I during storage and then form I melts and melts at 141 °C. The diffraction pattern changes again while cooling, which suggests there is recrystallisation.

**Fig. 9:**
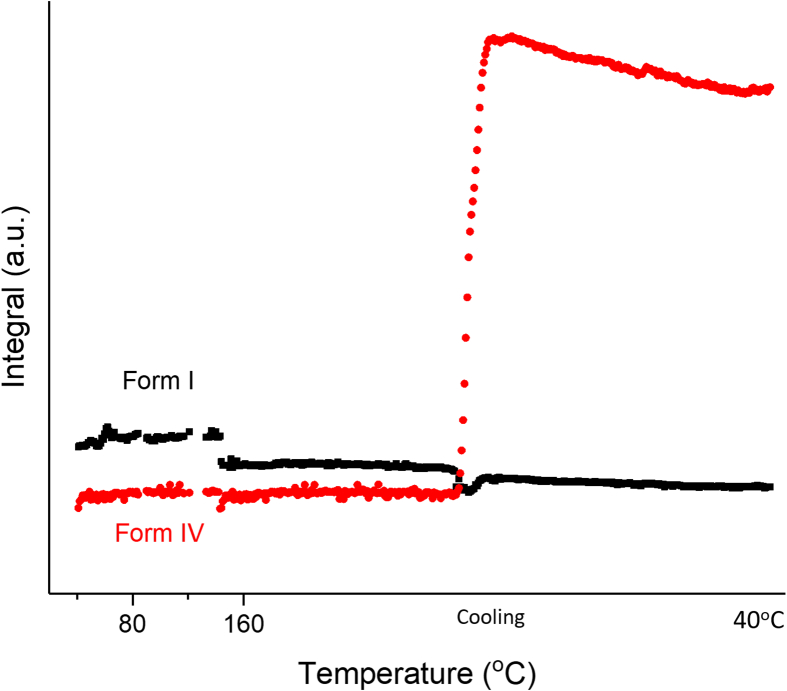
Plot of integrated total diffracted intensity for FFA form IV and form I as a function of temperature in a FFA: trehalose 1:5 w/w blend.

The patterns collected at 87 °C during heating and 45 °C during cooling were examined individually. The FFA in the recorded patterns is found to be present as form I and form IV respectively. Details of the refinement data are shown in Fig. S5 and Table S4. The phase fraction of FFA at 87 °C is 27%, significantly lower than its molar ratio (FFA: 86%, trehalose: 14%). This suggests that only a small amount of FFA crystallised at 87 °C, while most of remains in the amorphous state. Batch refinements were carried out to determine the amount of each FFA polymorph present in the sample as a function of temperature (see Fig. 9). FFA in the sample exists as form I until the temperature reaches 144 °C, after which FFA melts. When the temperature begins to reduce, form IV crystallises from the melt.

### 3.5 FTIR

Drug-polymer interactions were studied by FTIR. Spectra were recorded for FFA and trehalose and compared with mixtures prepared as for DSC-XRD analysis and heated to 120 °C (1:2, 3:2 and 5:1 *w*/w), 100 and 120 °C (1:1 w/w). All the samples were heated in the DSC and then removed for FTIR characterisation.

**Fig. 10:**
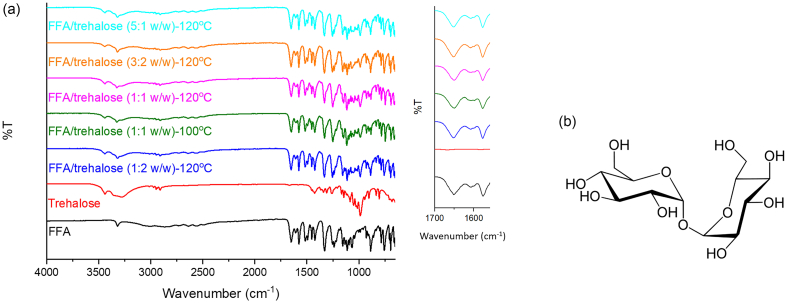
(a) FTIR spectra for FFA, trehalose and their mixtures after recrystallisation, and (b) the chemical structure of trehalose.

According to the DSC-XRD studies discussed above, upon heating FFA/T blends at 1:2, 3:2 and 5:1 w/w generate form I (at 120 °C), while the 1:1 w/w sample recrystallises into forms I and II at 100 °C and converts to form I at 120 °C. FTIR spectra are presented in Fig. 10a. FFA shows a characteristic peak due to the stretching of the secondary amine N—H at 3318 cm^−1^. The C=O and C=C vibrations are at 1654 and 1578 cm^−1^, respectively (Jabeen et al., 2012). For FFA-trehalose after heating, no shifts in the stretching of the secondary amine and vibration bands of carbonyl functional groups are seen. This result suggests that no H-bonds were formed between FFA and trehalose during heating. This is counter-intuitive, since trehalose has hydroxyl groups (Fig. 10b) which should form H-bonds with the carboxylic group of FFA. A lack of H-bonds could explain why FFA in the blends finally recrystallises into form I during heating, because there are no interactions to stabilise the metastable form IV. It should be noted however that the amine and carboxylate groups in all FFA polymorphs are heavily involved in FFA-FFA intermolecular H-bonding, and thus there is limited availability for them to interact with trehalose.

## 4 Discussion

During heat-cool cycles in the DSC, FFA-trehalose mixtures give different phase transition behaviour, dependent on the relative amount of trehalose present. In the DSC-XRD study (Table 1), all the mixtures had recrystallised to some extent upon storage, owing to the need to prepare the samples in advance of visiting the synchrotron. When the trehalose ratio is low (FFA: trehalose = 5: 1 *w*/w), FFA recrystallises into form I, while in the other formulations FFA form IV is generated upon storage. Before characterisation at Diamond, the samples were stored at 6 °C in a fridge, which suggests that a higher trehalose content can stabilise FFA form IV at low temperature.

**Table 1:** A summary of the phase transitions observed in FFA-trehalose mixtures obtained by cooling in DSC.

**Ratio**		**Polymorphic forms**			
**FFA**	**Trehalose**	**Initial**	**Phase transition**		**Cooling process**
			**I**	**II**	
1	2	Form IV	Form I	/	Form I
1	1	Form IV	Form I, Form II	Form I	Form I
3	2	Form IV	Form I	/	Form I
5	1	Form I	/	/	Form IV

During heating, phase transitions from form IV to other FFA forms occurred at around 80 °C for the FFA trehalose mixtures, as has also been reported for pure FFA (Pang et al., 2022). It thus appears that trehalose does not stabilise FFA form IV upon heating. FFA from the mixtures converted into form I, as was seen for pure FFA at high temperatures, with the transition temperatures being the same in all cases. Thus, it appears that trehalose cannot stabilise metastable forms of FFA at elevated temperature. During cooling, FFA in the mixture with a low trehalose content (FFA: trehalose = 5: 1 *w*/w) recrystallised into form IV, while the FFA in the other formulations converted into form I. Since FTIR results suggest no H-bonds were formed between FFA and trehalose, this leads to the hypothesis that trehalose may not affect the relative phase stability of FFA, but it may alter the activation energy for the transformation of amorphous FFA to form I and form IV. Therefore, when the trehalose content is high enough, the activation energy for amorphous FFA to convert to form I will be low enough for this transition without passing through metastable form IV.

The literature shows polymers are able to stabilise FFA form IV or amorphous phases during heating (Pang et al., 2022); however, in FFA-trehalose blends growth of form I is observed during heating. This agrees with the FTIR results, which show a lacking of H-bond formation between FFA and trehalose. Hence, there are no interactions which can help to stabilise metastable forms during heating.

## 5 Conclusions

Undertaking heat-cool cycles with FFA-trehalose mixtures prepared at different trehalose contents revealed that the phase transitions occurring varied with the composition of the blend. During storage, vitrified blends with low trehalose content (FFA: trehalose = 5:1 *w*/w) recrystallised into form I FFA, while higher trehalose content led to FFA form IV being generated after storage. When heated in the DSC, all FFA trehalose mixtures ultimately led to form I FFA, though form II was also seen with a 1:1 *w*/w ratio. Upon a second cooling cycle, mixtures with low trehalose content (FFA: trehalose = 5:1 *w*/w) transformed into form IV, while FFA with higher trehalose content converted to form I. Different trehalose ratios to FFA ratios thus demonstrate the ability to influence the polymorphic transitions of FFA.

## Author contributions

Conceptualization, Y·P. S.G., G.R.W.; methodology, Y.P., O.V.M.; formal analysis, Y.P.; investigation, Y.P., O.V.M.; resources, S.G., G.R.W.; data curation, Y.P, G.R.W.; writing—original draft preparation, Y.P.; writing—review and editing: Y.P., S.G., O.V.M., G.R.W.; visualization, Y.P.; supervision, S.G., G.R.W.; project administration, G.R.W.; funding acquisition, S.G., G.R.W. All authors have read and agreed to the published version of the manuscript.

## Declaration of Competing Interest

The authors declare that they have no known competing financial interests or personal relationships that could have appeared to influence the work reported in this paper.

## Data Availability

Data will be made available on request.

## Appendix A Supplementary data

Supplementary material Image 1
