# Supplementary material for: Polymorphic transitions in flufenamic acid-trehalose composites
Source: Int J Pharm X. 2023 Jul 23;6:100200. doi: 10.1016/j.ijpx.2023.100200 (PMC10410518; doi:10.1016/j.ijpx.2023.100200)
Supplement: Supplementary file 1 — Supplementary material [file mmc1.docx]

## **Supplementary information for**

## **Polymorphic transitions in flufenamic acid-trehalose composites**

Yuying Pang^a^, Simon Gaisford^a^, Oxana V. Magdysyuk^b^ and Gareth R. Williams^a*^

^a^ UCL School of Pharmacy, University College London, 29-39 Brunswick Square, London, WC1N 1AX, United Kingdom

^b^ Diamond Light Source, Harwell Science and innovation Campus, Didcot, Oxfordshire, OX11 0DE, United Kingdom

Correspondance email: [g.williams@ucl.ac.uk](mailto:g.williams@ucl.ac.uk)

Table S1. Refinement parameters for FFA in XRD patterns collected during reheating and cooling a 1: 2 w/w FFA/T blend. The starting models were taken from the CSD (FFA form I: FPAMCA11, form IV: FPAMCA15, trehalose β: SUJBOR02).

| **Form** | **IV** | **I** | **I** |
| --- | --- | --- | --- |
| **Temperature (^o^C)** | 78 | 115 | 75 (cooling) |
| **Space group** | *P*-1 | *P*21/*c* | *P*21/*c* |
| **a/ Å** | 8.7917(36) | 12.632(1) | 12.597(0) |
| **b/ Å** | 11.914(2) | 7.9136(3) | 7.8991(4) |
| **c/ Å** | 19.948(7) | 12.962(0) | 12.931(1) |
| **α/ ^o^** | 81.416(32) | 90 | 90 |
| **β/ ^o^** | 82.199(39) | 90.149(3) | 95.134(3) |
| **γ/ ^o^** | 74.003(26) | 90 | 90 |
| **Cell volume (Å)** | 1941.46 | 1290.53 | 1281.53 |
| **R_wp_** | 9.8962 | 5.7196 | 8.9413 |
| **Phase fraction^a^** | 58% | 31% | 44% |

^a^The representative error of the phase fractions cannot be calculated because of the graininess of the sample. Phase fraction refers to the percentage of a specific FFA form in the total crystalline material.

Table S2. FFA refinement parameters obtained during reheating and cooling a FFA/T blend (1: 1 w/w). The starting models were taken from the CSD (form I: FPAMCA11, form II: FPAMCA17, form IV: FPAMCA15, trehalose β: SUJBOR02).

| **Form** | **IV** | **I** | **II** | **I** | **I** |
| --- | --- | --- | --- | --- | --- |
| **Temperature (^o^C)** | 47 | 91 | | 128 | 68 (cooling) |
| **Space group** | *P*-1 | *P*2_1_/*c* | *P*2_1_/*c* | *P*2_1_/*c* | *P*2_1_/*c* |
| **a/ Å** | 8.5882(28) | 12.610(1) | 11.410(0) | 12.657(0) | 12.592(0) |
| **b/ Å** | 11.724(4) | 7.9091(5) | 10.476(1) | 7.9286(3) | 7.8864(3) |
| **c/ Å** | 19.858(5) | 12.945(1) | 11.880(1) | 12.964(1) | 12.937(1) |
| **α/ ^o^** | 80.528(27) | 90 | 90 | 90 | 90 |
| **β/ ^o^** | 82.761(25) | 95.104(4) | 113.34(1) | 95.176(3) | 94.940(5) |
| **γ/ ^o^** | 73.703(28) | 90 | 90 | 90 | 90 |
| **Cell volume (Å)** | 1886.46 | 1285.92 | 1304.12 | 1297.65 | 1279.99 |
| **R_wp_** | 4.3946 | 4.2565 | | 5.0096 | 6.0108 |
| **Phase fraction^a^** | 73% | 35% | 23% | 61% | 63% |

^a^The representative error of the phase fractions cannot be calculated because of the graininess of the sample. Phase fraction refers to the percentage of a specific FFA form in total crystalline material.

Table S3. FFA refinement parameters obtained when reheating and cooling a FFA/T blend (3: 2 w/w). The starting models were taken from the CSD (form I: FPAMCA11, form IV: FPAMCA15, trehalose β: SUJBOR02).

| **Form** | **IV** | **I** | **I** |
| --- | --- | --- | --- |
| **Temperature (^o^C)** | 71 | 120 | 63 (cooling) |
| **Space group** | *P*-1 | *P*2_1_/*c* | *P*2_1_/*c* |
| **a/ Å** | 8.7615(29) | 12.638(1) | 12.529(2) |
| **b/ Å** | 11.892(1) | 7.9144(6) | 7.8849(9) |
| **c/ Å** | 20.083(9) | 12.963(1) | 12.873(3) |
| **α/ ^o^** | 80.728(32) | 90 | 90 |
| **β/ ^o^** | 81.667(38) | 95.141(8) | 94.904(11) |
| **γ/ ^o^** | 73.651(18) | 90 | 90 |
| **Cell volume (Å)** | 1973.86 | 1286.83 | 1254.68 |
| **R_wp_** | 7.8748 | 3.7253 | 8.5611 |
| **Phase fraction^a^** | 71% | 50% | 80% |

^a^The representative error of the phase fractions cannot be calculated because of the graininess of the sample. Phase fraction refers to the percentage of a specific FFA form in total crystalline material.

Table S4. FFA refinement parameters obtained during reheating a FFA/T blend (5: 1 w/w). The starting models were taken from the CSD (form I: FPAMCA11, form IV: FPAMCA15, trehalose β: SUJBOR02).

| **Form** | **I** | **IV** |
| --- | --- | --- |
| **Temperature (^o^C)** | 87 | 45 |
| **Space group** | *P*2_1_/*c* | *P*-1 |
| **a/ Å** | 12.496(10) | 8.7365(32) |
| **b/ Å** | 7.9051(8) | 11.898(2) |
| **c/ Å** | 12.932(14) | 19.867(4) |
| **α/ ^o^** | 90 | 81.745(22) |
| **β/ ^o^** | 95.218(66) | 82.905(29) |
| **γ/ ^o^** | 90 | 74.655(21) |
| **Cell volume (Å)** | 1272.14 | 1963.11 |
| **R_wp_** | 6.9581 | 8.5434 |
| **Phase fraction^a^** | 27% | 86% |

^a^The representative error of the phase fractions cannot be calculated because of the graininess of the sample. Phase fraction refers to the percentage of a specific FFA form in total crystalline material.

Figure S1. Overlay of DSC profiles of FFA/trehalose blends heated from 0 to 150 ^o^C at 10 ^o^C/min and then cooled to 0 ^o^C. Exo up.

**(a)**

**(b)**

**(c)**

**(d)**

Figure S2. Rietveld refinement against diffraction patterns recorded for a 1:2 w/w FFA/T blend at a) 78 °C, with tick marks showing the positions of allowed reflections of FFA form IV (upper), trehalose β (lower); b) 115 ^o^C, with tick marks showing the position of allowed reflections of FFA form I (upper), trehalose β (lower); c) 121 ^o^C (during cooling), with tick marks showing the positions of allowed reflections of trehalose β; d) at 75 ^o^C (during cooling), where tick marks show the positions of allowed reflections of FFA form I (upper), trehalose β (lower).


**(a)**

**(b)**


**(c)**

**(d)**

**(e)**

Figure S3. Rietveld refinements for diffraction patterns recorded for 1:1 w/w FFA/ T at a) 47 °C (tick marks show the positions of allowed reflections of FFA form IV (upper), trehalose β (lower)); b) 91 ^o^C (tick marks show the position of allowed reflections of FFA form I (upper), form II (middle), trehalose β (lower)); c) 128^o^C (tick marks show the positions of allowed reflections of form I (upper), trehalose β (lower)); d) 157 ^o^C (tick marks show the positions of allowed reflections of trehalose β); and, e) at 68 ^o^C (on cooling; tick marks show the positions of allowed reflections of FFA form I (upper), trehalose β (lower)).

**(a)**

**(b)**


**(c)**

**(d)**

Figure S4. Rietveld refinements against diffraction patterns recorded for FFA/T 3:2 w/w at a) 71 °C (tick marks show the positions of allowed reflections of FFA form IV (upper), trehalose β (lower)); b)120 ^o^C (tick marks show the position of allowed reflections of FFA form I (upper), trehalose β (lower)); c) 160 ^o^C (tick marks show the positions of allowed reflections of trehalose β); d) 63 ^o^C (cooling) (tick marks show the positions of allowed reflections of FFA form I (upper), trehalose β (lower)).


**(a)**

**(b)**

**(c)**

Figure S5. Rietveld refinement against diffraction patterns recorded for a 5:1 w/w mixture of FFA and trehalose a) at 87°C, where tick marks show the positions of allowed reflections of FFA form I (upper), trehalose β (lower); b) at 160^o^C, with tick marks showing the positions of allowed reflections of trehalose β; c) at 45^o^C (cooling), where tick marks show the positions of allowed reflections of FFA form IV (upper), trehalose β (lower).
